# Family and socioeconomic risk factors for undernutrition among children aged 6 to 23 Months in Ibadan, Nigeria

**DOI:** 10.11604/pamj.2014.17.161.2389

**Published:** 2014-03-05

**Authors:** Eme Owoaje, Oluwadolapo Onifade, Adeyimika Desmennu

**Affiliations:** 1 Department of Community Medicine, University of Ibadan, Ibadan, Oyo State, Nigeria; 2 Department of Health Promotion and Education, University of Ibadan, Ibadan, Oyo State, Nigeria

**Keywords:** Child undernutrition, family characteristics, socio-economic factors

## Abstract

**Introduction:**

Child undernutrition is a major public health problem in Nigeria and other Sub-Saharan African countries. However, few analytical studies have quantified the role of risk factors. This study was conducted to determine the socio-economic and family related risk factors for undernutrition among children in Ibadan, Nigeria.

**Methods:**

A case-control study was conducted among children100 cases and 200 controls aged 6-23 months. A semi-structured interviewer- administered questionnaire was used to obtain information on socio-economic status, infant feeding practices of the mothers, children's immunization status and recent episodes of common childhood illnesses. Bivariate and multivariate analyses were conducted to identify the risk factors.

**Results:**

On bivariate analysis, the maternal factors associated with undernutrition were maternal level of education below secondary level, monthly income below $20 and polygamous marriage. Socio-economic factors significantly associated with malnutrition were residence in a high density area, family accommodation in a single room apartment and family weekly expenditure on food below $55. Children's characteristics associated with child malnutrition included incomplete immunization for age, recent episodes of diarrhoea and acute respiratory infection. The significant risk factors on multivariate analysis were maternal monthly income <$20, monthly household food expenditure <$55, residence in a one room apartment, higher birth order and incomplete immunization of the child.

**Conclusion:**

The multiplicity of risk factors identified is indicative of the need for a multidisciplinary approach in developing preventive strategies child undernutrition.

## Introduction

Child undernutrition is global public health problem especially in low-income and middle income countries [1, 2] and it is an underlying cause in more than a third of all infant and child deaths annually[3]. In many countries the effects of under-nutrition and infection have a synergistic impact on child mortality [2]. For the millions of surviving children, long term malnutrition results in disability from the irreversible physical and mental health effects of poor dietary intake. Severe malnutrition adversely affects the healthy development of children, impedes cognitive growth and ultimately hinders economic productivity in adulthood [4, 5]. Africa and Asia have more severe burdens of under-nutrition [6]. In Africa, almost two out of five children are stunted giving an estimated total of 60million children. Nigeria alone accounts for 11 million of these children [4]. Recent reports from the Nigerian National Demographic Health Survey 2009 indicate that 4% of children under the age of five years were stunted, while 23% and 14% were underweight and wasted respectively[7].

Several studies have reported factorscontributory to childhood malnutrition in Nigeria. The major risk factors identified include family and socioeconomic variables, educational attainment of mothers, difficult family conditions such as unemployment, parental separation and mother being pregnant with another child. Other risk factors are sub-optimal nutrition due to lower breastfeeding rates or early cessation of breastfeeding, lack of food and poor environmental conditions [8–11]. Most of these have been identified by descriptive cross-sectional studies which documented the prevalence of childhood malnutrition and the associations with selected risk factors. However, few recent studies have quantified the risk attributable to various important social and economic risk factors for childhood undernutrition using an analytical study design. Information obtained using such methodology is crucial for identifying groups to be prioritized in targeted interventions. This paper reports the findings of a study of the socioeconomic and environmental risk factors for undernutrition among children attending a secondary health care facility in Ibadan, south west Nigeria.

## Methods

### Study location

Oni Memorial Children's Hospital is a 60-bed paediatric specialist facility in Ibadan, southwest Nigeria. The hospital provides preventive and curative health services for children from varying socio-economic classes. It also serves as a referral hospital for primary health care centres and other secondary health centres in the state. On average, about 2,000 children are seen in the hospital out-patient clinic severy month.

### Study design

This was a case control study among children 6 to 23 months of age. Nutritional status was defined based on the American National Center for Health Statistics (NCHS) standards.[12] All the new cases of underweight children i.e. with weight-for-age<2 standard deviations from the median weight-for-age of the reference population the eligible age group who presented at the Nutrition Clinic within the period of data collection (6 weeks) were recruited as cases until the desired number was attained. Controls were children in the target age group who were not underweight i.e. weight-for-age values equal or above 2 standard deviations from the median weight-for-age of the reference population, who presented at the Immunization clinic and the Infant welfare clinic. Children who had underlying conditions which could lead to the faltering of growth, such as chronic diseases, congenital malformations and chromosomal abnormalities were excluded from the study.

### Sample size estimation

Using the formula for case control studies, [13] The sample size was calculated based on the proportion of Nigerian mothers with no formal education(50% based on findings of the 2003 NDHS), a ratio of one case to two controls, power of 80%,significance level of 5% (95% confidence interval) and an odds ratio of two. A minimum sample size of 99 and 198 for cases and controls respectively was obtained, hence 100 cases: 200 controls. Each case was matched by age and sex with two controls.

### Data Collection

An interviewer administered semi-structured questionnaire was used to interview the mothers of the children who were selected to participate in the study. Data was collected on the family social and demographic characteristics, household characteristics, infant feeding practices, immunization history and recent episodes of acute illnesses in the children. The questionnaire was administered in English or Yoruba depending on the respondents’ preference.

### Measurements

**Weight**: weight measurements were taken to the nearest 0.1kg using Salter 914WHLKR baby scale. The scale was checked before each weighing to ensure that the mark returned to zero. The children were weighted without clothes on and weights were taken in kilograms. Each child was weighed twice.

**Length**: measurement of supine or recumbent length was taken to the nearest 0.1cm using a portable calibrated board, the child's growth chart. The sole of the baby's feet were held firmly against the wall at the zero point while the length was marked off on the chart at the crown of the head.

### Data Management

Data was entered and analyzed using SPSS version 15. The Nutritional module of CDC/WHO Epi-Info 6.0 software was used to convert the anthropometrical indices weight for age, height for age and weight for height Z- scores based using the WHO/CDC 2000 reference standards. The wealth index developed was on the principle components analysis of household assets. The questions used to establish the wealth index included household access to electricity, radio or television; household ownership of bicycle, motorcycle or car; type of material of used for flooring the house; number of rooms in the house; main source of drinking water; type of toilet facility. Principle components analysis was used to derive wealth index quintiles and the ranking of these quintiles were used to represent household wealth. Pearson's Chi square test was conducted to determine associations between categorical variables. Bivariate analysis was conducted; odds ratios with95% confidence intervals were calculated to determine the risk factors for childhood undernutrition. Multivariate analysis was conducted to assess the contribution of each risk factor.

## Results

### Children's Characteristics

One hundred underweight cases and 200 controls were enrolled in the study (Table 1). A significantly higher proportion of the cases were of a higher birth order (>4) compared to the controls (20.0% versus 5.5%, OR1.98 95%CI 1.22-3.20), they were also more likely to be living with only one parent or a non-parent relative (16.0% versus 7.5%, OR1.42 95%CI 0.97-2.06). Overall, the rate of exclusive breastfeeding was low in both groups but the cases were less likely to have been exclusively breastfed for six months (23% versus 58% respectively, OR 1.3; 95% CI 1.2-1.52). Furthermore, the mothers of the cases were less likely to have breastfed them on demand compared with those of the controls (21% versus 5.5%, OR 2.0 95% 1.2-3.3). Assessment of the immunisation status of the children revealed that the cases were more likely to have not received the complete immunisation required for their age compared to the controls, (91.1% versus 28.0%,OR 2.0 95%CI 2.00-3.04). The reported history of illness in the 30 day period prior to the study was more common among cases than controls. The cases were more likely to have experienced episodes of diarrhoea (29.0% versus 9%, OR 4.1, 95% CI 2.2-7.9 and symptoms of acute respiratory tract infections (31.0% versus 15.0%, OR 2.54, 95% CI 1.4-4.5).

**Table 1:** Characteristics of cases and controls

Characteristic	Cases N=100 n (%)	Controls N=200 n (%)	P Value	Odds Ratio (95% CI)
**Age (months)**				
6-11	43 (43.0)	86 (43.0)	0.5	
12-17	34 (34.0)	70(35.0)		
≥ 18	23 (23.0)	44 (22.0)		
**Sex**				
Male	32 (32.0)	64 (32.0)	0.55	
Female	68 (68.0)	136 (68.0)		
**Birth Order**				
>4	20(20.0)	11(5.5)	0.00	1.98 (1.22-3.20)
≤4	80(80.0)	189(94.5)		
**Living with Both parents**				
No	16(16.0)	15(7.5)	0.027	1.42(0.97-2.06)
Yes	84(84.0)	185(92.5)		
**Exclusively breastfed for 6months**				
No	77(77.0)	116(58.0)		
Yes	23(23.0)	84(42.0)	0.001	1.30 (1.12-1.52)
**Breastfed on demand**				
No	21(21.0)	11(5.5)		
Yes	79(79.0)	189(94.5)	0.01	2.0(1.2-3.3)
**Immunisation status for age**				
Incomplete	91(91.0)	56(28.0)	<0.005	2.47(2.00-3.04)
Complete	9(9.0)	144(72.0)		
**Recent illness**				
Yes	50(50.0)	68(34.0)	0.009	1.94(1.2-3.16)
No	50(50.0)	132(66.0)		
**Recent history of diarrhoea**				4.1(2.2-7.9)
Yes	29(29.0)	18(9.0)	<0.005	
No	71(71.0)	182(91.0)		
**Recent history of ARI**				
Yes	31(31.0)	30(15.0)		
No	69(69.0)	170(85.0)	0.002	2.54(1.4-4.5)

### Parents’ Characteristics

The socio-demographic characteristics of the children's parents and selected living conditions are shown in Table 2. A significantly higher proportion of mothers of the cases were aged below 25 years (28.0%) compared to 18.0% of the mothers of controls. The mothers of the cases were more likely to have been uneducated or received only primary school education (35% versus 26%, OR 3.6 95% CI 2.0-6.4) and they were also more likely to be engaged in unskilled or partially skilled occupations (68% versus 50.5%, 1.26 95% CI 1.1-1.5). Six percent of the mothers of the cases were single while none of the mothers of the controls were single. Among those who were married polygamy was commoner among the families of the cases (22.3%) compared to those of the controls (4.0%) (OR 6.90 95%CI 2.92-16.27). A significantly higher proportion of the fathers of the cases (15%) had received no education or only primary school education compared to only 2.5% of the fathers of the controls (p<0.05, OR 6.88 95%CI 2.4-19.54). However, there were no significant differences in the fathers’ occupational groups.

**Table 2:** Family and household characteristics by study status

Characteristics	Cases N=100 n(%)	Controls N=200 n(%)	P Value	Odds Ratio (95%CI)
**Mother's Age (years)**				
<25	28(28.0)	36(18.0)	.034	1.77(1.0-3.12)
≥25	72(72.0)	264(82.0)		
**Mother's education**				
Below secondary school	35(35.0)	26(26.0)	<0.005	3.60(2.01-6.44)
Secondary school and above	65(65.0)	174(74.0)		
**Mother's occupation**				
Unskilled/Partially Skilled	68(68.0)	101(50.5)	0.003	1.26 (1.1-1.5)
Skilled/Managerial/Professional	32(32.0)	99(49.5)		
**Mother's income**				
<$20	45(45.0)	31(15.5)	<0.005	4.46(2.57-7.72)
≥$20	55(55.0)	169(84.5)		
**Father's education**				
Below secondary school	15(15.0)	5(2.5)	<0.05	6.88(2.4-19.54)
Secondary school and above	85(85.0)	195(97.5)		
**Father's occupation**				
Unskilled/Partially skilled	55(55.0)	119(59.5)	0.02	1.2(1.0-1.44)
Skilled/Managerial/Professional	45(45.0)	81(40.5)		
**Marital Status of mother**				
Single	6(6.0)	0(0.0)		3.13(2.64-3.69)
Married	94(94.0)	200(100.0)	0.001*	
**Type of Marriage N=294**				
Monogamous	73(77.7)	192(96.0)	<0.05	
Polygamous	21(22.3)	8(4.0%)		6.90(2.92-16.27)
Living arrangement				
Living together all week	79(79.0)	165(82.5)		0.79(0.43-1.46)
Living separately during the week/ mostly	21(21.0)	35(17.5)	0.28	
**Number of people in home**				
<4	71(71.0)	172(86.0)	<0.05	11.93(5.45-26.12)
≥4	29(29.0)	28(14.0)		
**Living in a one room apartment**				
Yes	36(36.0)	9(4.5)	<0.01	11.94(5.86-25.50)
No	64(64.0)	191(95.5)		
**Household monthly food expenditure**				
<N8000 ($55)	46(46.0)	17(8.5)	<0.05	6.27(2.21-17.83)
≥N8000($55)	54(54.0)	183(91.5)		
**Use of sanitary waste disposal system**				
Yes	58(58.0)	189(94.5)	<0.05	12.44(6.03-25.66)
No	42(42.0)	11(5.5)		
**Wealth Index quintile**				
Lowest 3 quintiles	66(66.0)	114(57.0)	<0.05	
4^th^-5^th^ Wealth quintiles	34(34.0)	86(43.0)		1.13(0.96-1.32)

* Fishers exact correction

In terms of maternal income, a significantly higher proportion of case mothers (45.0%) reportedly earned <$20 per month compared to 15% of the mothers of the controls, (OR 4.46 95% CI 2.57-7.72). Similarly, monthly food expenditure was less than $55 in a significantly higher proportion of the households of the cases (46.0%) compared with only 8.5% in the households of the controls (OR 6.27 95% CI 2.21-17.83). The families of the cases were also more likely to be living in one bedroom households (36% versus 4.5%, OR 11.93 95% CI 5.4 -26.12)

### Predictors for Underweight

On the multivariate analysis, the predictors for underweight were: the child being the fourth or higher birth order (AOR 4.5 95% CI 1.33-15.5); family residence in a one room apartment (AOR 6.17 95% CI 1.72-22.1), mother's monthly income of less than $20 (AOR 6.16 95% CI 2.0-18.97); household monthly expenditure of less than $55 (AOR 6.54 95% CI 1.30- 32.78) and incomplete immunization in the child (AOR 9.61 95% CI 3.6 - 25.52)(Table 3).

**Table 3:** Risk factors for undernutrition

Risk Factor	Odds Ratio	95% CI	P-value
**Birth order**			
≥4	4.5	1.33-12-15.2	0.015
**Residence**			
One room	6.17	1.72-22.1	0.005
**Mother's monthly income**			
<$20	6.16	2.00-18.97	0.02
**Household expenditure on food per month**			
< $55	6.54	1.30-32.78	0.02
**Immunisation status**			
Incomplete	9.61	3.60-25.52	<0.05

## Discussion

The findings of this case control study confirm the role of various factors in the occurrence of undernutrition. Most of the factors were related to socio-economic status, though paternal income was not obtained, maternal income and household monthly food expenditure were significant risk factors. Furthermore, children whose mothers had less than secondary level education had a higher risk of being undernourished than those whose mothers had been educated beyond primary school level. This is keeping with findings from studies conducted in low and middle income countries including Nigeria [7, 11, 14–16]. However, in this study maternal education lost its significance on logistic regression. This may be due to the fact that other socio-economic factors are more important role than maternal education in promoting child nutrition.

The strong association between sub-optimal breastfeeding practices and undernutrition has been shown by other researchers [17, 18]. The rate of exclusively breastfeeding in both groups of children was low but significantly lower among the cases in this study. Similarly, the children who were cases were less likely to have been breastfed on demand. However these significant associations were not risk factors for undernutrition on multivariate analysis. As reported in other studies, recent episodes of acute illnesses such as diarrhoea and acute respiratory infection were also significantly higher among the cases [14, 16, 19]. However, in this study these risk factors lost their significance on multivariate analysis.

Immunisation is a cost effective intervention for the prevention of childhood diseases and consequent undernutrition and complete immunisation has been identified as an indicator of access to modern health care[16]. Incomplete complete immunization was observed to predict a nine fold risk of undernutrition among the cases. This finding is suggestive of the important role inadequate access to formal health facilities and modern health care services due to cost, distance or ignorance could have on childhood undernutrition in this environment. This relationship needs to be explored by other researchers. Children from homes with more than four children had a four- fold higher risk of developing malnutrition compared to those from homes with ≤4 children. This is consistent with the findings of other researchers who have identified large family size as a risk factor for under-nutrition [16, 20]. It has been reported that the quality of childcare in the presence of many siblings is impaired due to the limited time available for the mother to devote to the care of each child[21]. Furthermore, breastfeeding of the younger child is often compromised, while the care of the older children maybe deficient thus contributing to malnutrition[16]. This further reiterates the important role of family planning and limiting family size in improving child health.

A key factor underlying most of the determinants of undernutrition is poverty. In this study, residence in a one room apartment, low maternal income and household expenditure on food were socioeconomic indicators which remained significant risk factors of under-nutrition on the regression analysis. These findings have been corroborated by other studies [10, 14, 22, 23]. Generally, individuals residing in poor households are unable to achieve food security and less likely to access modern health facilities [24].

## Conclusion

This study demonstrated multiplicity risk factors for child related family socioeconomic status and income. Higher birth order of and incomplete immunization status were prominent factors for the children. The varieties of risk factors identified in this study emphasise the need for a multidisciplinary approach to address the issues of child undernutrition.
